# Identification of new protein-coding genes with a potential role in the virulence of the plant pathogen *Xanthomonas euvesicatoria*

**DOI:** 10.1186/s12864-017-4041-7

**Published:** 2017-08-16

**Authors:** Ulrike Abendroth, Norman Adlung, Andreas Otto, Benjamin Grüneisen, Dörte Becher, Ulla Bonas

**Affiliations:** 1 Institute for Biology, Department of Genetics, Martin-Luther-Universität Halle-Wittenberg, D-06099 Halle, Germany; 2 Institute for Microbiology, Department of Mass Spectrometry, Ernst-Moritz-Arndt-Universität, D-17487 Greifswald, Germany; 3 Department of Psychiatry and Psychotherapy, Martin-Luther-Universität Halle-Wittenberg, D-06097 Halle, Germany

**Keywords:** *Xanthomonas*, Proteogenome, Ortho proteogenomic, Genome re-annotation, Translational start sites, T3SS, T4SS, T6SS

## Abstract

**Background:**

Bacteria of the genus *Xanthomonas* are economically important plant pathogens. Pathogenicity of *Xanthomonas* spp. depends on the type III-secretion system and additional virulence determinants. The number of sequenced *Xanthomonas* genomes increases rapidly, however, accurate annotation of these genomes is difficult, because it relies on gene prediction programs. In this study, we used a mass-spectrometry (MS)-based approach to identify the proteome of *Xanthomonas euvesicatoria* (*Xe*) strain 85–10 also known as *X. campestris* pv. *vesicatoria*, a well-studied member of plant-pathogenic *Xanthomonadaceae*.

**Results:**

Using different culture conditions, MS-datasets were searched against a six-frame-translated genome database of *Xe*. In total, we identified 2588 proteins covering 55% of the *Xe* genome, including 764 hitherto hypothetical proteins. Our proteogenomic approach identified 30 new protein-coding genes and allowed correction of the N-termini of 50 protein-coding genes. For five novel and two N-terminally corrected genes the corresponding proteins were confirmed by immunoblot. Furthermore, our data indicate that two putative type VI-secretion systems encoded in *Xe* play no role in bacterial virulence which was experimentally confirmed.

**Conclusions:**

The discovery and re-annotation of numerous genes in the genome of *Xe* shows that also a well-annotated genome can be improved. Additionally, our proteogenomic analyses validates “hypothetical” proteins and will improve annotation of *Xanthomonadaceae* genomes, providing a solid basis for further studies.

**Electronic supplementary material:**

The online version of this article (doi:10.1186/s12864-017-4041-7) contains supplementary material, which is available to authorized users.

## Background

Since the first sequenced genome of phage ϕX174 in 1978 the number of sequenced genomes has steadily increased due to the development of new and efficient sequencing methods [1, 2]. Today, a major challenge is not the sequencing of new genomes, but the correct annotation of as many genes as possible, the basis for detailed functional analyses. Coding sequences (CDS) are usually annotated using gene prediction software such as Glimmer, Prodigal, Genemark and Easygene [3–6]. The high coding capacity (up to 90%) of bacterial, viral and archaeal genomes [3] require a high accuracy of gene prediction programs. An important quality parameter of prediction software is the sensitivity, i.e., how many of the known genes in a well-characterized genome are predicted [3]. One example is the 99% sensitivity of the first version of Glimmer (2.0) [7]. With respect to protein-coding genes, a major challenge is the correct prediction of the translation start sites (TSS) because homology often decreases in the vicinity of the TSS [8]. Gene annotation quality can be improved by the integration of transcriptome and, more importantly, proteome data using a mass-spectrometry based approach. Proteogenomics integrates shot-gun proteome information into the genome annotation process [9], thereby directly mapping MS-spectra to the six possible open reading frames. This helps to validate predicted protein-coding genes and improves genome annotation. Refinement of a given genome annotation can then be extended to related species using comparative genomics.

Our lab studies the Gram-negative γ-proteobacterium *Xanthomonas euvesicatoria* strain 85–10 (*Xe*), also termed *X. campestris* pv. *vesicatoria* [10, 11], which causes bacterial spot disease on pepper and tomato plants [12]. The genus *Xanthomonas* comprises economically important pathogens that together infect a wide range of crop plant species [13]. *Xe* enters the plant tissue via natural openings, e.g., stomata, or wounds and multiplies locally in the intercellular space [14]. Pathogenicity of *Xe* relies on the type III-secretion system (T3SS), which is encoded by the chromosomal *hrp* (hypersensitive response and pathogenicity)-gene cluster [15, 16] and translocates bacterial effector proteins (T3E) directly into the plant cell [17]. Expression of the T3SS components is induced during infection and in special minimal media (e.g., XVM2 [18]). The key regulator HrpG, an OmpR-type response regulator is activated by unknown plant signals and controls the expression of a large *hrp*-regulon, including many T3E [19]. The isolation of a point mutation in *hrpG* (termed hrpG*), which renders the HrpG protein constitutively active, was key for the analysis of the T3SS and the identification of new virulence factors [20].

The genome sequence of our model *Xe* strain 85–10 was published in 2005 [12] and has a G + C-content of 64.5%. Besides the 5.18 Mb chromosome, there are four plasmids, pXCV2, pXCV19, pXCV38 and pXCV183 (1.8 kb, 19 kb, 38 kb and 182.5 kb, respectively) [12]. In the original annotation, 4726 genes for proteins and functional RNAs were predicted. This number did not include yet the 24 genes for small non-protein coding RNAs (sRNAs) which were recently identified by an RNA-seq approach [21]. The latter approach also revealed unusually long 5′-UTRs for a number of T3E genes suggesting incorrectly annotated TSS. One confirmed example is the T3E XopD whose N-terminus had to be extended by 215 amino acids (aa) [22].

Here, we propose a re-annotation of the *Xe* 85–10 genome using proteogenomic data obtained in a large-scale experiment. This is the first study to propose a *Xe* genome refinement, which can be extended to other economically important bacterial genera.

## Results

### Proteogenomic analysis of *Xe* 85–10

**Fig. 1:**
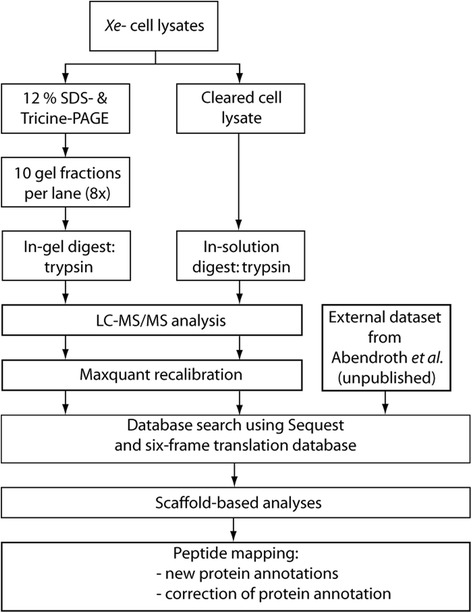
Experimental workflow of the proteogenomic analysis of *Xe*. The *Xe* strains 85–10, 85* and 85-10Δ*sX13* were grown in NYG, Minimal medium A pH 7 and XVM2, respectively, at 30 °C until OD_600_ of either 0.5 (exponential), 0.8 (early stationary) or 1.2 (stationary). Proteins extracted from *Xe* 85–10, 85-10Δ*sX13* and 85* cell lysates were separated by 12% SDS PAGE and Tricine PAGE. Gel fractions and cell lysate were digested by trypsin. Samples were analyzed by LC–MS/MS. A database search against a six-frame translation database of *Xe* 85–10 was performed. Peptides were mapped to the genome of *Xe* using a TBlastN-based approach. The dataset from Abendroth et al. is originally a comparative study between *Xe* strains 85–10 and 85-10Δ*sX13* and is based on the original genome annotation. The MS spectra of this dataset were also searched against the six-frame database

The overall goal of this study was to identify as many proteins as possible that are expressed in the *Xe* strain 85–10 and its derivative 85*. 85* carries a point mutation in *hrpG* which renders the expression of the T3SS and effector genes constitutive in minimal media and complex medium NYG [20]. For MS analyses bacteria were grown to exponential and stationary phase, respectively, in three different media: NYG, minimal medium A (MA) pH 7 and XVM2. MA and XVM2 media induce the T3SS and T3E genes [18]. Bacterial cells were ruptured by French press, and the lysates analysed as shown in the flow-sheet (Fig. 1, for details see Methods). MS/MS analyses revealed 845,925 spectra which were assigned to peptide sequences using Sequest and an in silico translated six-frame database of *Xe* 85–10. The rationale behind this was the aim to cover all annotated coding sequences but also possible CDS missed in the original genome annotation [12]. Peptides were mapped to 2588 CDS thus covering 54.7% of the *Xe* 85–10 genome. Please note that 2500 CDS map to the chromosome (Additional file 1) and the remaining 88 CDS to the four *Xe* 85–10 plasmids. Given 1684 hypothetical CDS (termed hypothetical, or putative secreted or membrane proteins) in the originally annotated *Xe* 85–10 genome, we validated the expression of 764 CDS on the protein level (45%) (Additional file 2).

**Fig. 2:**
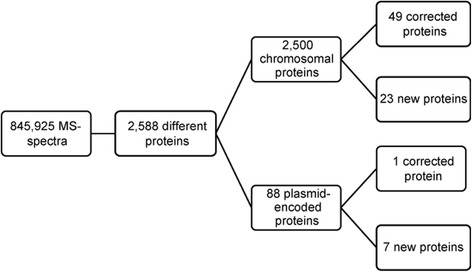
Overview of proteins identified in the proteogenomic analysis of *Xe*

**Fig. 3:**
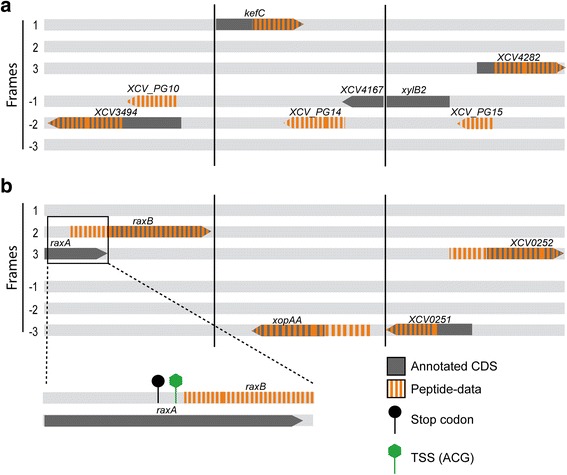
Schematic overview of chromosomal regions with detected new and corrected protein-coding genes. **a** Examples of three new protein-coding genes detected by proteogenomics, *XCV_PG10*, *XCV_PG14* and *XCV_PG15*. **b** Examples of three corrected protein-coding genes detected by proteogenomics with a close-up of the *raxA/B* region. All six reading frames are shown. *Grey*: annotated CDS; orange dashes represent peptide-data detected by MS/MS. *Black* circle represents a stop codon; the green hexagon represents the possible translation start codon of *raxB*

Mapping of the peptides to the six-frame genome database revealed (i) 50 protein-coding regions with a longer N-terminal region than annotated and (ii) 30 new genes (Fig. 2 and Tables 1 and 2, for additional information see Additional file 3). If the annotation would be corrected based on the new data, 32 genes would overlap now with previously annotated CDS, e.g., the newly identified protein-coding genes *XCV_PG10* and *XCV_PG15* (Fig. 3a), and *raxB* and *XCV0251* (Fig. 3b), for which the original annotation likely has to be revisited since MS-data for the raxB protein point toward a new TSS and raxA spectra are missing.

**Table 1:** Summary of incorrectly annotated genes

ID^a^	Gene	Chromosomal/plasmid position	Detected length (aa)	Annotated length (aa)
Erroneously annotated CDS on *Xe* chromosome				
0056329	*XCV0252*	292545..293186	214	103
0056337	*dcp2*	295098..297215	706	697
0162384	*XCV0352*	403872..404171 c	100	98
0107340	*hrpD6*	469724..470224 c	167	85
0002631	*xopD*	486784..488823	757	545
0057941	*hsdS1*	576612..577958	449	419
0003072	*trpC*	584455..585336	294	265
0106644	*XCV0529*	595352..597223 c	624	532
0030344	*XCV0537*	602135..603208	358	308
0133900	*XCV0557*	624664..625527 c	281	247
0161114	*XCV0564*	632247..633056 c	270	144
0003611	*XCV0612*	693670..694671	334	326
0105852	*pheC*	736187..737158 c	324	266
0032290	*XCV0855*	974414..975196	261	260
0059889	*XCV0861*	983577..986486	968	660
0061379	*XCV1116*	1247631..1248212	194	193
0034580	*raxB*	1401839..1404616	926	718
0156885	*XCV1265*	1423680..1424456 c	259	208
0007939	*XCV1378*	1558348..1558917	190	162
0156077	*XCV1397*	1577580..1578143 c	188	185
0036069	*dapD*	1672904..1674070	389	285
0036594	*grpE*	1761374..1762582	403	172
0154666	*hutU*	1889784..1891697 c	638	555
0099525	*XCV1716*	1935113..1936231 c	373	272
0010368	*XCV1807*	2036752..2038401	550	497
0098447	*XCV1885*	2132024..2133079 c	352	188
0067505	*XCV2100*	2394132..2395619	469	306
0125297	*flgG*	2310361..2311182 c	274	261
0040728	*exbB2*	2584091..2584648	186	183
0150414	*XCV2312*	2645805..2646305 c	167	150
0122465	*XCV2513*	2837767..2838294 c	176	89
0094634	*cydD*	2857544..2859259 c	572	570
0094553	*argB*	2874455..2876008 c	518	426
0122245	*dksA*	2884537..2885304 c	256	147
0093090	*gumE*	3162956..3164299 c	448	433
0120648	*infC*	3173992..3174486 c	165	156
0091857	*XCV2971*	3378614..3379942 c	443	375
0073386	*cheB2*	3442416..3444254	613	369
0019248	*XCV3212*	3657508..3659298	597	518
0020234	*XCV3377*	3862711..3863067	119	103
0075628	*XCV3419*	3905421..3907445	675	557
0114862	*xpsM*	4216381..4217121 c	247	217
0114852	*xpsK*	4218136..4219170 c	345	301
0114582	*rmlD*	4282300..4283133 c	278	273
0086369	*XCV3785*	4369448..4371499 c	684	616
0085725	*rpoD*	4490900..4492780 c	627	625
0078964	*rho*	4539894..4541690	599	420
0053728	*guaA*	4966625..4967413	266	256
0053999	*XCV4380*	5042765..5043472	236	222
Erroneously annotated CDS on *Xe* plasmid				
0166278	*XCVd0050*	56630..57289	220	217

^a^ Number of the corresponding six-frame-database entry

c chromosomal position on the minus strand

For detailed information see Additional file 3

**Table 2:** Novel genes identified in this study

ID^a^	Name	Neighboring genes	Detected protein length (aa)	Plausible protein length (aa)^b^
New CDS found on *Xe* chromosome				
0136836	*XCV_PG01*	*XCV0062-XCV0063*	242	256
0055942	*XCV_PG02*	*XCV0209-XCV0210*	114	116
0028571	*XCV_PG03*	*XCV0214-XCV0215*	241	306
0056540	*XCV_PG04*	*XCV0282-XCV0283*	77	98
0065083	*XCV_PG05*	*parE-pyrG*	25	59
0094126	*XCV_PG06*	*XCV2618-XCV2619*	76	107
0043902	*XCV_PG07*	*XCV2723-XCV2724*	42	70
0089084	*XCV_PG08*	*XCV3389-virB6*	111	161
0020369	*XCV_PG09*	*XCV3391-XCV3392*	73	141
0143360	*XCV_PG10*	*XCV3494*	47	117
0087222	*XCV_PG11*	*XCV3657-xpsD*	59	99
0022971	*XCV_PG12*	*XCV3783-XCV3784*	150	191
0050568	*XCV_PG13*	*rsmC-XCV3801*	131	157
0112004	*XCV_PG14*	*kefC-XCV4167*	125	148
0111304	*XCV_PG15*	*xylB2-XCV4282*	65	‡
0081693	*XCV_PG16*	*XCV4416-XCV4417*	112	141
New CDS found on *Xe* plasmids				
0175626	*XCV_PG17*	*after XCVa0002*	53	60
0173148	*XCV_PG18*	*before XCVc0001*	34	109
0172926	*XCV_PG19*	*tnpR-XCVc0009*	74	76
0174118	*XCV_PG20*	*XCVc0025-XCVc0026*	123	138
0169438	*XCV_PG21*	*XCVd0054-XCVd0055*	92	132
0166803	*XCV_PG22*	*XCVd0124-XCVd0125*	107	129
New CDS found antisense to annotated CDS				
0152041	*XCV_PG23*	*anti-XCV2096†*	30	39
0122029	*XCV_PG24*	*anti-XCV2593†*	258	258
0049655	*XCV_PG25*	*anti-xadA1†*	1552	1597
0080326	*XCV_PG26*	*anti-XCV4209†*	200	203
0166979	*XCV_PG27*	*anti-XCVd0155†*	41	51
0013218	*XCV_PG28*	anti-glk1*	143	162
0008300	*XCV_PG29*	anti-XCV1454*	508	‡
0007106	*XCV_PG30*	anti-gcvP*	820	837

^a^ Number of the corresponding six-frame-database entry

† no MS-data for the annotated protein detected

* MS-data for the annotated protein detected

^b^ Protein length till the next plausible translation start site (ATG, GTG, TTG)

‡ No plausible translation start site (ATG, GTG, TTG) between detected peptide and the next upstream stop codon

For detailed information see Additional file 3

### Protein coding genes with longer N-terminal regions in *Xe*

**Fig. 4:**
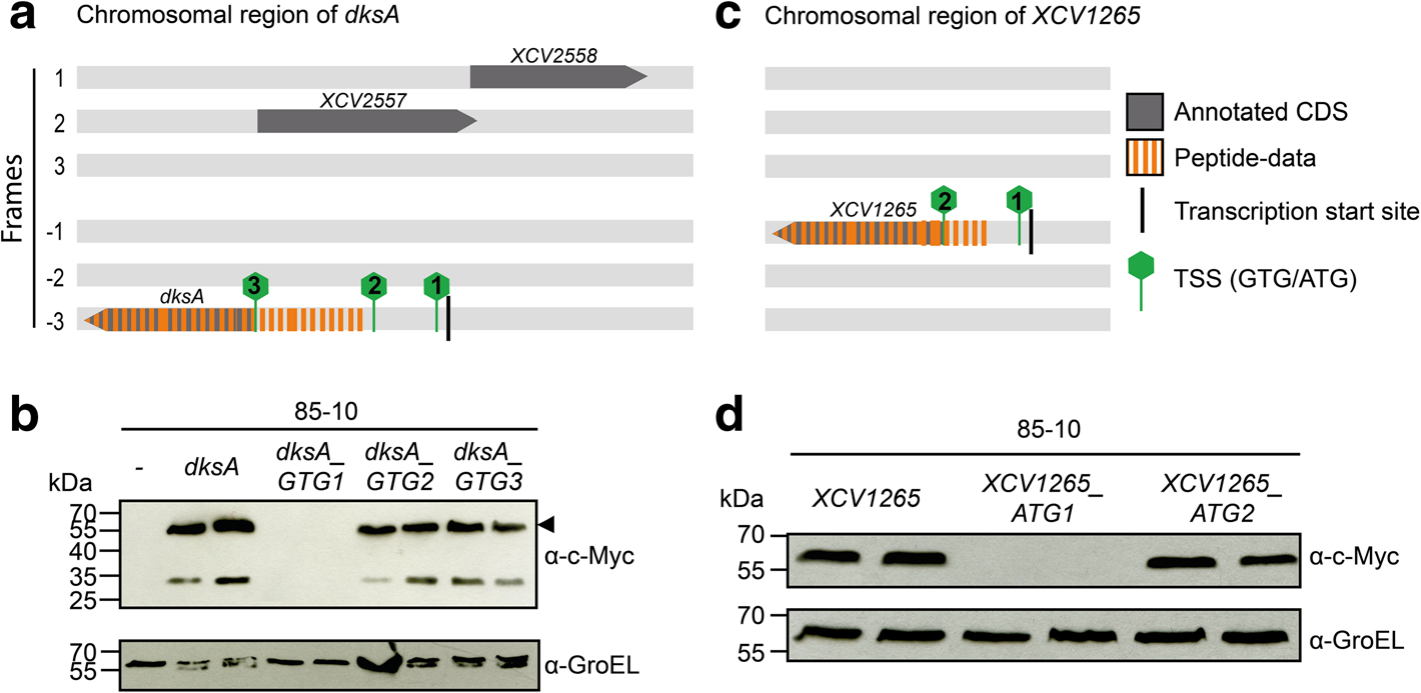
Gene organization of the *dksA* and *XCV1265* regions. **a** and **c** *dksA* and *XCV1265* loci of *Xe*. All six reading frames are shown. Grey: annotated CDS; orange dashes: peptide-data detected by MS/MS; green hexagons: possible translation start codons of *dksA* and *XCV1265*. **b** and **d** Analysis of potential translation start codons of DksA and XCV1265. Total protein extracts of *Xe* 85–10 containing pBRM-P(*dksA*), pBRM-P(*dksA_GTG1*), pBRM-P (*dksA_GTG2*), pBRM-P (*dksA_GTG3*), pBRM-P (*XCV1265*), pBRM-P (*XCV1265_ATG1*), pBRM-P (*XCV1265_ATG2*) or an empty vector (−) were separated by 12% SDS PAGE and analyzed by immunoblotting using a c-Myc-specific antibody. As loading control, membranes were reacted with a GroEL-specific antibody. Experiments were repeated at least twice with similar results

The prediction of the most likely TSS is a critical point in genome annotations. In GC-rich genomes, ~60% of genes might have a incorrectly annotated TSS [23]. To identify erroneously annotated TSS we searched for peptides located upstream of and in the same frame as a previously annotated TSS. Out of the 50 longer genes 49 are encoded on the chromosome and one on pXCV183, the largest plasmid (Table 1). Among the longer genes is *dksA*, which now largely overlaps with *XCV2557*, encoded on the opposite strand (Fig. 4a) and not represented by any peptides in this study. Thus, we propose to delete *XCV2557.* That this appears to be justified is based on a previous transcriptome study which revealed a transcription start site for *dksA* overlapping with *XCV2557* [21]. Given the *dksA* transcription start site and peptides covering this genomic region our new data suggest two possible TSS (Fig. 4a). Site-directed mutagenesis of the annotated and the possible TSS revealed that protein translation most likely starts at the first GTG (Fig. 4b). Using expression constructs whose expression is driven by the native promoter, we observed not only that the first GTG is used, but also a processed variant of DksA. We also experimentally analyzed TSS of *XCV1265*, encoding a D-alanyl-D-alanine carboxypeptidase. For *XCV1265*, peptide data suggest a TSS further upstream than in the annotation, which was confirmed by site-directed mutagenesis (Fig. 4c and d).

Another example of a longer than previously thought gene is *dcp2* for which a peptide overlapping the annotated GTG TSS suggests an ATG start codon further upstream. This is supported by ortho-proteogenomic analysis of other members of the *Xanthomonadaceae* (Additional file 4). Similarly, our peptide data and an ortho-proteogenomic analysis indicate that *exbB2* is incorrectly annotated (Additional file 4). Surprisingly, given the 64% G + C content in *Xe*, we found that for *infC* obviously the codon ATT is used as TSS. Here, we detected peptides further upstream of the previously annotated TSS. Additional sequence analyses showed that the only possible TSS is an ATT, because there is no alternative start codon (common TSS: ATG, GTG, TTG) between the last peptide-covered sequence and the stop codon (Additional file 4). *InfC* is well-analyzed in other bacteria, e.g., *E. coli,* where the same TSS codon is used [24, 25].

### Identification and verification of novel protein-coding genes

Intriguingly, our *Xe* proteomic approach identified 30 new genes encoding mostly small proteins with an average size of 191 amino acids (aa), ranging from 25 to 820 aa (Table 2). Among the small proteins is sX6, which was first assumed to act as sRNA but encodes a protein [21], which we could verify in our data. *Xe* harbours the 1852 bp plasmid pXCV2, which was thought to encode two protein-coding genes [26]. In this study, we detected peptides mapping to a third CDS which is located between position 1673 and 114 and encodes a protein of 60 aa (Additional file 5). Most new proteins have no annotated counterpart in other *Xanthomonas* genomes. However, 15 of the 30 protein-coding genes are conserved on the DNA level (Blast output: at least 80% coverage and 80% identity) suggesting that the corresponding proteins are also produced in other xanthomonads.

We predicted functional domains in the newly identified proteins. Interestingly, *XCV_PG01*, located between *XCV0062* and *XCV0063,* encodes a putative serine/threonine phosphatase of the 2C family, which was previously overlooked. Furthermore, we identified a putative YecR-like lipoprotein, *XCV*_PG06, which was recently annotated in *X. oryzicola* [27]*.* A special case is *XCV_PG30*, which is encoded antisense to *gcvP*, predicted to encode a metal-dependent RNase. Both corresponding proteins are represented by peptides in this study. However, most new proteins lack known functional domains.

**Fig. 5:**
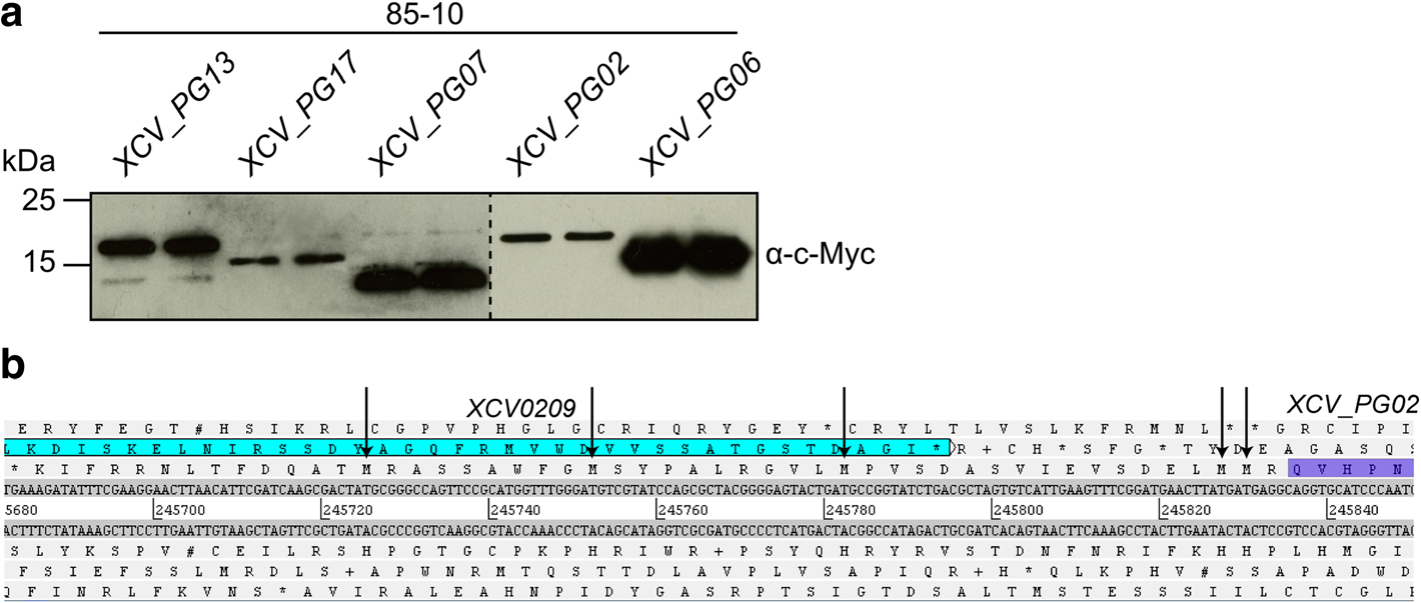
Validation of five new protein-coding genes. **a** Detection of the protein synthesis of new *Xe* proteins. Total protein extracts of *Xe* 85–10 containing pBRM-P (*XCV_PG13*), pBRM-P (*XCV_PG17*), pBRM-P (*XCV_PG07*), pBRM-P (*XCV_PG02*) or pBRM-P (*XCV_PG06*) grown in NYG were separated by 15% SDS PAGE and analyzed by immunoblotting using a c-Myc-specific antibody. **b** Gene organization of the *XCV_PG02* locus. *XCV0209* and *XCV_PG02* are highlighted. Arrows represent possible translation start codons of *XCV_PG02*

To validate the MS-data experimentally by an independent method, the expression of five new protein-coding genes was tested. An important criterion for the selected genes was the knowledge of the exact transcription start sites [21]. C-terminal c-Myc tagged expression constructs under the control of the native promoter were generated in pBRM-P and transformed into *Xe* 85–10. As shown in Fig. 5a, all tested new CDS expressed proteins of expected molecular mass. In case of *XCV*_PG02 there are five possible TSS (Fig. 5b). As the correct TSS cannot be deduced from the immunoblot it needs to be determined by alternative methods.

### The putative T6SS of *Xe* has no virulence function in standard virulence assays

**Fig. 6:**
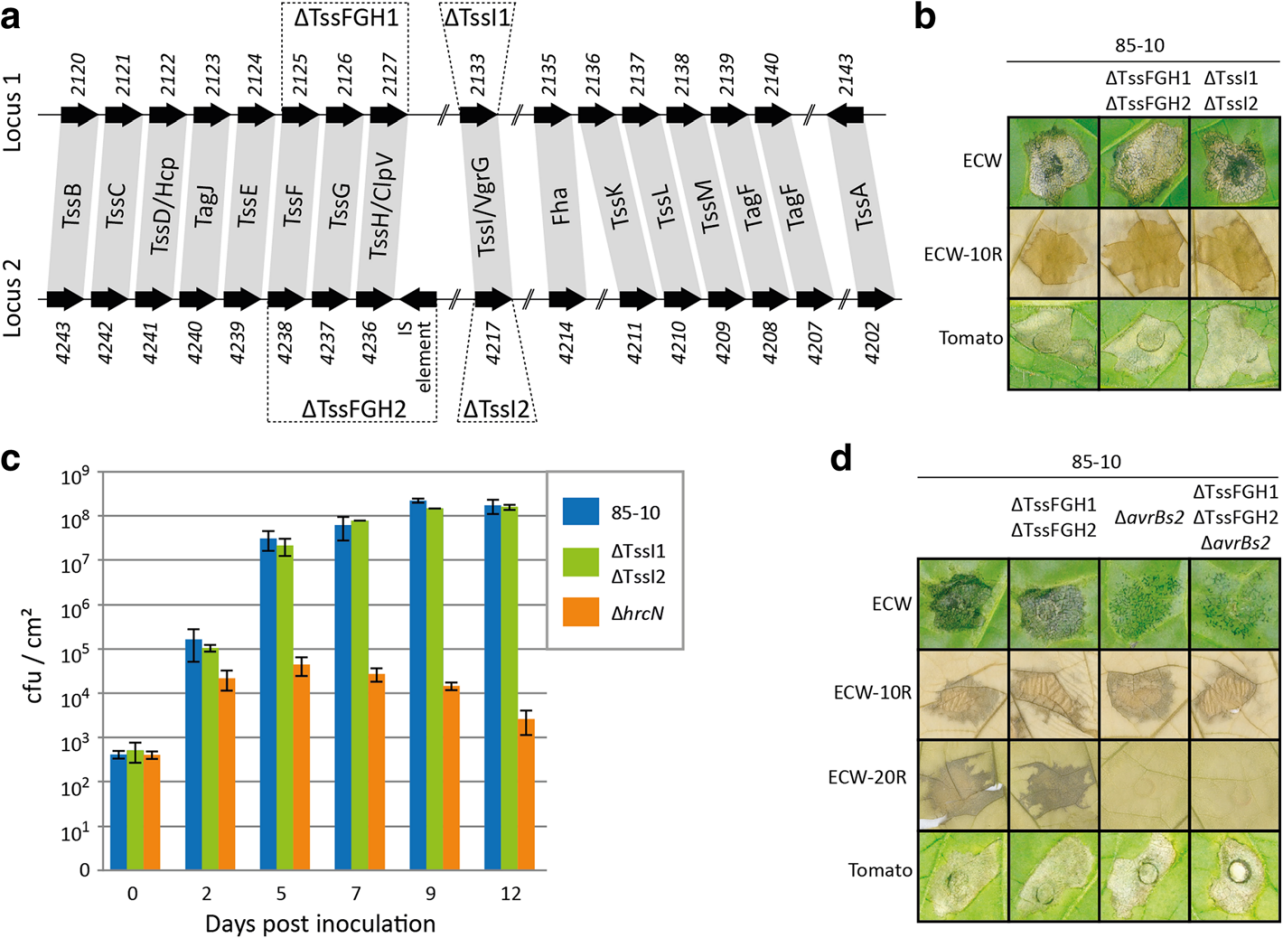
Deletion of conserved T6SS components has no effect on *Xe* virulence. **a** Schematic representation of the two genomic T6SS loci in *Xe*. Gene numbers and commonly used gene names of T6SS components identified in *Xe* 85–10 are given. Dashed lines mark genes deleted in this study. **b** *Xe* strains 85–10, 85-10ΔTssFGH1ΔTssFGH2 and 85-10ΔTssI1ΔTssI2 were inoculated into susceptible pepper plants (ECW), resistant pepper plants (ECW-10R) and susceptible tomato plants with an OD_600_ of 0.1. Phenotypes were documented 7 days post inoculation (7 dpi, ECW), 2 dpi (ECW-10R) and 8 dpi (tomato). **c** *Xe* strains 85–10, 85-10ΔTssI1ΔTssI2 and 85-10Δ*hrcN* (T3SS mutant) were inoculated in ECW plants with an OD_600_ of 4 × 10^−5^. Bacterial multiplication in leaves was monitored for 12 days. **d** *Xe* strains 85–10, 85-10ΔTssFGH1ΔTssFGH2, 85-10Δa*vrBs2* and 85-10ΔTssFGH1ΔTssFGH2Δ*avrBs2* were inoculated with an OD_600_ of 0.1 into leaves of pepper plants (ECW, ECW-10R, ECW-20R) and tomato plants. Phenotypes were documented 6 dpi (ECW), 2 dpi (ECW-10R, ECW-20R) and 9 dpi (tomato). Leaves of ECW-10R and ECW-20R plants were bleached in EtOH for better visualization of cell death reactions. Experiments were repeated twice with similar results

Since the bacteria were grown in T3SS-inducing conditions, we expected to detect peptides corresponding to known virulence factors, i.e., T3SS components and T3E. Our MS-analysis identified 69% of T3E and 84% of structural and regulatory T3SS proteins (Table 3 and Additional file 6). Furthermore, 10 of 11 known Xps type II-secretion system (T2SS) components and 3 of 5 known substrates [28, 29] were detected (Table 3 and Additional file 6). *Xe* also encodes the Xcs T2SS, which in contrast to the Xps T2SS does not contribute to virulence [28]. No components of the Xcs T2SS were detected in our study. Various components of type IV-secretion systems (T4SS) [12] and type VI-secretion systems (T6SS) are encoded in *Xe* [30], but it is unknown if these putative secretion systems are functional in *Xe*. In order to identify a potential virulence function of putative T4SS and T6SS, we analyzed whether components were detected in our MS-data. Ten out of the 18 predicted components of the Vir-type T4SS were detected in our MS/MS-data, but no component of the Icm/Dot-type T4SS (Table 3 and Additional file 6). In addition, we analyzed two loci in the *Xe* genome, each encoding 15 conserved T6SS components (Fig. 6a). Only the T6SS component TssH/ClpV was detected in our MS-data (Table 3 and Additional file 6).

**Table 3:** Summary of MS/MS-data on secretion systems

Secretion system	# of detected / known proteins
Tat and Sec-dependent secretion	15/19
T1SS	4/4
T2SS – Xcs-type	0/12
T2SS – Xps-type	10/11
T2SS – substrates	3/5
T3SS	21/25
T3E	25/36
T4SS – *vir*-type	10/18
T4SS – *icm*-type	0/15
T5-autotransporter	3/4
T6SS – locus 1	0/16
T6SS – locus 2	1/16

For detailed list see Additional file 6 (Excel file)

To test whether the putative T6SS contribute to virulence of *Xe,* deletion mutants were generated. On one hand, we deleted the TssI/VgrG-encoding gene of both loci (*xcv2133* and *xcv4217*, termed TssI1 and TssI2) and on the other hand, TssF-, TssG- and TssH/ClpV-encoding genes of both loci (*XCV2125*-*XCV2127*, termed TssFGH1 and *XCV4236*-*XCV4238*, termed TssFGH2) were deleted (Fig. 6a). In characterized T6SS these components are essential for function [31]. The generated mutants, *Xe* 85-10ΔTssFGH1ΔTssFGH2 and *Xe* 85-10ΔTssI1ΔTssI2 were inoculated into pepper and tomato plants to test for virulence. The deletion mutants behaved like wild type, i.e., disease symptoms in susceptible plants and HR induction in resistant pepper plants (Fig. 6b). We also monitored the *in planta* growth of *Xe* 85-10ΔTssI1ΔTssI2 in comparison to *Xe* 85–10 in leaves of susceptible pepper plants; there were no significant differences (Fig. 6c). As a control, a strain without functional T3SS (*Xe* 85-10Δ*hrcN*) was used [32]. As expected, multiplication of *Xe* 85-10Δ*hrcN* was strongly reduced (Fig. 6c). Next, we additionally deleted *avrBs2* in *Xe* 85-10ΔTssFGH1ΔTssFGH2. The T3E AvrBs2 is recognized in ECW-20R pepper plants [33] and is a conserved virulence factor in xanthomonads [34]. Deletion of *avrBs2* renders *Xe* less virulent and helps to analyze subtle virulence effects when other genes are mutated. However, disease symptoms induced by *Xe* 85-10ΔTssFGH1ΔTssFGH2Δ*avrBs2,* in pepper ECW and tomato plants were comparable to those induced by *Xe* 85-10Δ*avrBs2* (Fig. 6d). Taken together, deletion of conserved T6SS components did not affect virulence of *Xe* under the conditions tested.

## Discussion

Because of its economical relevance, *Xanthomonas* spp. are currently subject of intense sequencing efforts and more and more genomes are available [35]. Here, we demonstrate the importance of proteogenomics for a better characterization of these important plant pathogens. Analyses of a large MS-spectra data set of *Xe* 85–10 and 85*, cultivated in different media, identified 30 new genes and 50 incorrectly annotated CDS. The number of inaccuracies in the *Xe* genome annotation [12] is comparable to previous proteogenomic studies of other bacteria, e.g., *Yersina*, *Helicobacter*, *Mycobacterium*, *Rugeria* and *Deinococcus* [25, 36–39]. These studies led to the refinement of 4–41 new and 5–73 falsely annotated genes and reached overall genome coverage of 31–80%. Thus, our study (55%) falls into the average genome coverage. It is expected that the coverage will increase with the number of conditions tested, because proteins might be exclusively synthetized under specific conditions or below the detection limit. As our lab focusses on the analysis of proteins important for the host-pathogen interaction, we chose respective conditions, i.e., XVM2, minimal medium A pH 7 (MA 7) and *Xe* strain 85*. Identified peptides corresponded to 25 (69%) known T3E and 21 (84%) gene products of the *hrp*-gene cluster (Additional file 6). Eleven known T3E were not detected, which might be due to a low abundance within the bacterial cell. Two detected T3E, XopD und XopAA, are longer than annotated. The original *Xe* annotation stipulates that these effectors have a size of 545 aa (XopD) and 616 aa (XopAA) respectively. The MS/MS-data showed that XopD and XopAA are 215 aa and 72 aa, respectively, longer. These results are consistent with published data [22, 40] and corroborate the idea that unusually long 5′ untranslated regions in *Xe* T3E mRNAs might hint to incorrectly annotated transcription start sites [21, 22, 41]. These findings are of special importance as the N-terminal regions of T3E usually harbor the T3SS-secretion and -translocation signals [42]. The knowledge of the exact TSS is crucial for further studies of T3E.

Genome annotation inaccuracies are often due to CDS which are present in a small number of organisms, so that the power of comparative genomics is limited. Validation of longer proteins and newly identified proteins requires additional experimental evidence. In contrast to previous studies [39], we made an effort to validate novel proteins by Western blot analysis, using expression constructs controlled by the corresponding native promoter. The combined use of MS- and transcriptome data can suggest the existence of new genes, but detection of RNA alone is no proof for the existence of a CDS.

The use of the native promoter is only feasible for genes with a known transcription start site. Based on the transcriptome data of Schmidtke et al. (2012) seven expression constructs were created, and the synthesis of proteins was demonstrated by Western blot. For DksA and XCV1265 we detected signals corresponding to proteins with higher molecular weight than previously annotated and confirmed the respective TSS using site-directed mutagenesis. The transcription of *dksA* starts within *XCV2557* [21], encoded on the opposite strand (Fig. 4a). We propose that the previously annotated gene *XCV2557* next to *dksA*, for which peptide data are missing, does not exist, as it greatly overlaps with the newly proposed annotation of *dksA*. As for *dksA*, *Xe* harbors many other transcription start sites internal of protein-coding regions which might be a hint for annotation mistakes.

Furthermore, we propose that the *infC* translation does not start with the annotated ATG, but with ATT. An ATT start codon was also found in a proteogenomic study of *Deinococcus deserti* [25]. Interestingly, the translation of *infC* in *E. coli* is also initiated at an ATT start codon. In *E. coli*, the ATT start codon is used for auto-regulation of translation [24].

Besides the T3SS other secretion systems might play a role in *Xe* virulence. Our MS-analysis detected 56% of the components of a putative Vir-type T4SS. By contrast, components of the Xcs T2SS and the putative IcmDot-type T4SS were not detectable. Besides *Xe*, putative T4SS are encoded in many other xanthomonads, e.g., *X. axonopodis* pv. *citri* [43], *X. citri* pv. *citri* [44] and *X. campestris pv. campestris* [45]. The function of these systems has only been studied in a few cases. The Vir-type T4SS of *X. campestris* pv. *campestris* does not contribute to bacterial virulence [46] and transcription of Vir-type T4SS components is downregulated during infection of host plants in *X. citri* pv. *citri* 306 [47]. In addition, the T4SS was shown to act against other Gram-negative bacteria in a contact-dependent manner [44, 48].

T6SS are encoded in many xanthomonads [30] and the genome of *Xe* 85–10 harbors two T6SS loci, each encoding 15 conserved T6SS components. It is not unusual that bacterial genomes harbor different T6SS loci. For example, *Pseudomonas aeruginosa* encodes three independent T6SS [49] and *Burkholderia thailandensis* five independent T6SS [50]. Only a single T6SS component was detected in our MS-approach, suggesting that both T6SS of *Xe* might play a role under different conditions. Since a function of a T6SS in xanthomonads is elusive, we generated mutants in putative T6SS genes in *Xe*. However, the deletion mutants revealed no obvious role of the putative T6SS in the interaction with plants. The genes we deleted are predicted to result in a loss of function [31]. The T6SS of *Xe* might target other bacterial species, as shown for T6SS of *Vibrio cholerea* [51, 52], *Serratia marcescens* [53], *Salmonella* Typhimurium [54] and *P. aeruginosa* [55]. To answer this question was out of scope of this study and has to await further studies.

## Conclusions

Here, we describe that the well-annotated genome of *Xe* can be improved. Besides validation of “hypothetical” proteins, we discovered novel protein-coding genes and corrected the annotation of 50 genes. Proteins of particular biological interest, e.g., a serine/threonine phosphatase, putative secreted proteins and proteins containing domains of unknown functions were identified. Furthermore, the annotation of protein-coding genes which play a role in *Xanthomonas* virulence have been corrected, e.g., the T3SS-component HrpD6 and the T3SS-substrate XopAA. This proteogenomic analysis will improve annotations of *Xanthomonadaceae* genomes. Future studies of newly identified genes might unravel new virulence functions.

## Methods

### Bacterial strains and growth conditions

For bacterial strains, plasmids and oligonucleotides used in this study see Additional file 7. The *Xe* strains 85–10 [12, 56], 85* [20] and 85-10Δ*sX13* [57] were grown in NYG [58], Minimal medium A pH 7 [59] and XVM2 [18], respectively, at 30 °C until OD_600_ of 0.5 (exponential), 0.8 (early stationary) or 1.2 (stationary). Plasmids were introduced into *Xe* by tri-parental conjugation, using pRK2013 as helper plasmid [60, 61]. Antibiotics were added to a final concentration of: gentamycin, 15 μg/ml; rifampicin, 100 μg/ml, 100 μg/ml spectinomycin.

### Protein extraction and pre-separation

Cells were cracked in TE-buffer using three times French press. Cell debris and undissolved material were removed by centrifugation (15 min, 16,000×g, 4 °C). Protein concentrations were measured using the Bradford assay. 100 μg protein were precipitated over night with ice-cold acetone. Protein pellets were dissolved in 40 μl Laemmli-buffer, and 20 μl were subjected to 1-D-SDS PAGE (12% separation gel, 4% stacking gel). The gel was fixed overnight in 40% methanol and 10% acetic acid, and stained with colloidal Coomassie (20% Ethanol; 1.6% phosphoric acid; 8% ammonium sulfate; 0.08% Coomassie Brilliant Blue G-250).

### LC-MS/MS-measurements and data analysis

Lanes of the protein gel were cut into 10 slices of equal size and proteins were digested in gel by trypsin. The eluted peptides were subjected to LC-MS/MS-analysis on a Proxeon nLC 1000 coupled to an Orbitrap Elite mass spectrometer. In-house self-packed columns (i.d. 100 μm, o.d. 360 μm, length 150 mm; packed with 1.7 μm Aeris XB-C18 reversed-phase material (Phenomenex, Torrance, CA, USA) were loaded, then desalted with 10 μl buffer A (0.1% (*v*/v) acetic acid) at a maximum pressure of 750 bar. For LC-MS/MS-analysis, peptides were eluted using a nonlinear 80 min gradient from 1 to 99% buffer B (0.1% (*v*/v) acetic acid in acetonitrile) at a constant flow rate of 300 nl/min. Spectra were recorded in an Orbitrap Velos (Thermo Fisher Scientific, Waltham, MA, USA) at a resolution of *r* = 30,000 with lockmass correction activated. After acquisition of the Full-MS-spectra, up to 20 dependent scans (MS/MS) were performed according to precursor intensity by collision-induced dissociation fragmentation (CID) in the linear ion trap.

Data were analyzed by Sorcerer Sequest against a six-frame translated database of the whole *Xe* genome (protein database containing 175,698 (21,627 ≤ 6 aa) entries). The following search parameters were used: enzyme type, trypsin (KR); peptide tolerance, 10 ppm; tolerance for fragment ions, 1 Da; b- and y-ion series; a maximum of two modifications per peptide was allowed. Peptide and protein identifications were accepted with a false discovery rate (FDR) of maximal 0.4%, requiring a minimum of at least two unique peptides for protein identification and quantification.

A second data set was generated using MS/MS-data obtained from a comparative proteome experiment. The tryptic digests obtained from the 1-D-SDS PAGE gel pieces were subjected to reversed phase column chromatography (Waters BEH 1.7 μm, 100 μm i. d. × 100 mm, Waters Corporation, Milford, MA, USA) operated on a nanoACQUITY-UPLC (Waters Corporation, Milford, MA, USA). Peptides were concentrated and desalted on a trapping column (Waters nanoACQUITY UPLC column, Symmetry C18, 5 μm, 180 μm × 20 mm, Waters Corporation, Milford, MA, USA) for 3 min at a flow rate of 1 ml/min with 99% buffer A (0.1% acetic acid). Subsequently, the peptides were eluted and separated using a non-linear 80-min gradient from 5 to 60% ACN in 0.1% acetic acid at a constant flow rate of 400 nl/min. MS and MS/MS-data were obtained using the LTQ-Orbitrap mass spectrometer (Thermo Fisher Scientific, Waltham, MA, USA) equipped with a nanoelectrospray ion source. After a survey scan in the Orbitrap (*r* = 30,000) with the lockmass option enabled, MS/MS-data were recorded for the five most intensive precursor ions in the linear ion trap. Singly charged ions were not taken into account for MS/MS-analysis.

Data were analyzed by Sorcerer Sequest against the 6-frame database. The following search parameters were used: enzyme type, trypsin (KR); peptide tolerance, 10 ppm; tolerance for fragment ions, 1 Da; b- and y-ion series; a maximum of two modifications per peptide was allowed. Peptide and protein identifications were accepted with a false discovery rate below 1%, requiring a minimum of at least two unique peptides for protein identification and quantification.

### Peptide mapping and visualization

Identified peptides were mapped to the *Xe* genome using TBlastN [62], perfect and full length sequence matches were used. With this setup the best fit for the peptide to the *Xe*-DNA sequence was selected. The peptides were visualized in Artemis genome browser [63]. GFF files can be found in Additional file 8.

### Generation and mutation of expression constructs

For expression in *Xe*, protein coding sequences and the putative promoter region of *XCV_PG02*, *XCV_PG06, XCV_PG07*, *XCV_PG13*, *XCV_PG17*, *dksA* and *XCV1265* were amplified from genomic DNA of *Xe* 85–10 by PCR using oligonucleotides listed in Table 3 and cloned into pBRM-P [64] by Golden Gate cloning [65]. pBRM-P encodes a c-Myc epitope which is fused to the 3′ end of the insert.

To mutate possible TSS, site-directed mutagenesis was employed. For this, pBRM-P (*XCV1265*) or pBRM-P (*dksA*) were used as a template and PCR amplified using oligonucleotides harboring the desired mutation (Additional file 7). Primers carried a 5′ phosphate for subsequent circulation of amplicons.

### Protein analysis

To analyze the protein synthesis of XCV_PG02, XCV_PG06, XCV_PG07, XCV_PG13, XCV_PG17, DksA and XCV1265 *Xe* 85–10 bacteria with corresponding expression constructs were grown overnight in NYG medium until stationary phase. Protein extracts were analyzed by SDS-PAGE and immunoblotting using first an antibody specific for the c-Myc epitope (Santa Cruz Biotechnology, Dallas, TX, USA) and secondly, anti-GroEL (Enzo Life Sciences, Farmingdale, NY, USA). Secondary antibodies were horseradish peroxidase labeled anti-mouse or anti-rabbit antibodies (GE Healthcare, Chicago, IL, USA). Antibody reactions were visualized by enhanced chemiluminescence.

### Generation of deletion mutants

To generate deletion mutants, regions of about 1 kb flanking of the deleted sequences were amplified by PCR and cloned into the suicide vectors pOGG2 via Golden Gate cloning or pOKI via classical cloning (Table 3). An IS-element is encoded subsequently before *XCV4236* (TssH/ClpV), which was deleted together with *XCV4236*-*XCV4238* (TssFGH2). pOGG2 derivatives or pOKI (*avrBs2*) were conjugated into *Xe* and mutants were selected by PCR.

### Plant infection assays

Plants were grown in the greenhouse with 23 °C/25 °C day temperature (tomato/pepper) and 19 °C night temperature, 16 h of light and 40–60% humidity. For plant infection assays, *Xe* suspended in 10 mM MgCl_2_ were inoculated with a needleless syringe into leaves of the near-isogenic pepper (*Capsicum annuum*) cultivars ECW, ECW-10R or ECW-20R or tomato (*Solanum lycopersicum*) cultivar MoneyMaker [33, 66]. Pepper ECW is a commercial cultivar that has been used to introgress disease resistance genes and generate near-isogenic lines [33]. The tomato and pepper plants were grown as described before [56, 67].

## Additional files

Additional file 1: Proteogenomic identification of proteins in *Xe* 85–10. Overview of the *Xe* chromosome showing all annotated and MS-data based identified protein-coding genes. Black: annotated CDS plus strand, Red: annotated CDS minus strand, Black-Red: MS-data based identified CDS, Black serrates line: GC-content. (PNG 133 kb)

Additional file 2: Identification of all detected proteins and their annotated function. List of all detected annotated proteins in MS-data and their predicted functions. (XLSX 693 kb)

Additional file 3: Additional information to Tables 1 and 2 and all conditions and strains used in this study. The tables show additional information, e.g. predicted function, homology and transcription start site, of the new and falsely incorrectly annotated protein-coding genes and all conditions and strains used in this study. (XLSX 29 kb)

Additional file 4: Reannotation of *dcp2*, *exbB2*, *flgG* and *infC*. Multiple sequence alignment of *dcp2*, *exbB2*, *flgG* and *infC* homologs of *Xe*, *X. axonopodis* pv. *citrumelo* F1 (*XacF1*), *X. oryzae* pv. *oryzae* KACC10331 (*Xoo*), *X*. *oryzae* pv. *oryzicola* BLS256 (*Xoc*), *X*. *fuscans* subsp. *aurantifolii* ICPB 11122 (*Xfa*), *X. perforans* 91–118 (*Xp*). Green: experimentally detected by MS, underlined in red: annotated start codons, underlined in green: possible new start codon. (PNG 836 kb)

Additional file 5: pXCV2 carries a third CDS. Representation of pXCV2 plasmid of *Xe* 85–10. Grey arrows show position of annotated CDS and the red arrow indicates the position of the newly identified protein-coding CDS. (PNG 88 kb)

Additional file 6: Detection of proteins which are components or substrates of (potential) secretion systems. +: specific peptide detected, −: no specific peptide detected in MS-data. (XLSX 15 kb)

Additional file 7: Oligonucleotides, plasmids and strains used in this study. List of oligonucleotides, plasmids and strains used in this study. (DOCX 45 kb)

Additional file 8: GFF annotation file. GFF annotation file for artemis genome browser. (TXT 147 kb)

## Abbreviations

aa: Amino acid
CDS: Coding sequence
Da: Dalton
ECW: Early cal wonder
FDR: False discovery rate
GFF: General feature format
LC: Liquid chromatography
MA: Mini medium A
MS: Mass spectrometry
sRNA: Small RNA
T2SS: Type II secretion system
T3E: Type III effector
T3SS: Type III - secretion system
T4SS: Type IV - secretion system
T6SS: Type VI - secretion system
TSS: Translation start site
UTR: Untranslated region
