# Supplementary figures and images for: Identification of new protein-coding genes with a potential role in the virulence of the plant pathogen Xanthomonas euvesicatoria
Source: BMC Genomics. 2017 Aug 16;18:625. doi: 10.1186/s12864-017-4041-7 (PMC5559785; doi:10.1186/s12864-017-4041-7)

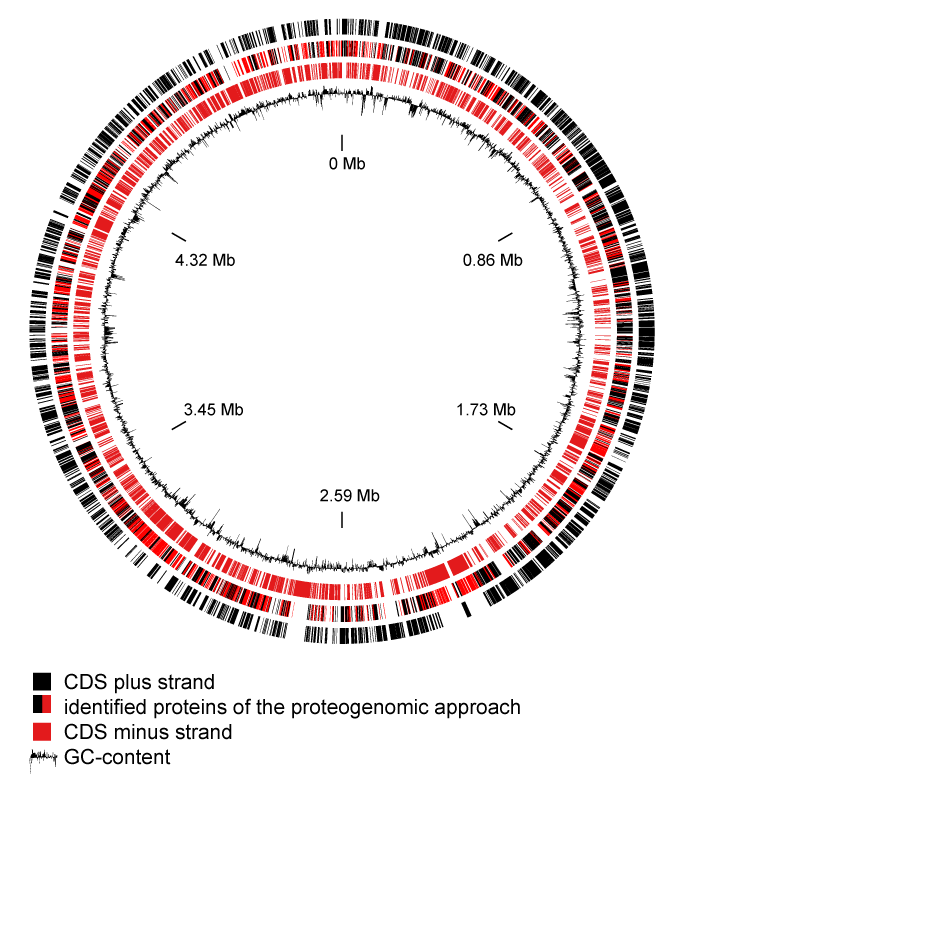

Supplement: Supplementary file 1 — Proteogenomic identification of proteins in Xe 85–10. Overview of the Xe chromosome showing all annotated and MS-data based identified protein-coding genes. Black: annotated CDS plus strand, Red: annotated CDS minus strand, Black-Red: MS-data based identified CDS, Black serrates line: GC-content. (PNG 133 kb) [file 12864_2017_4041_MOESM1_ESM.png]

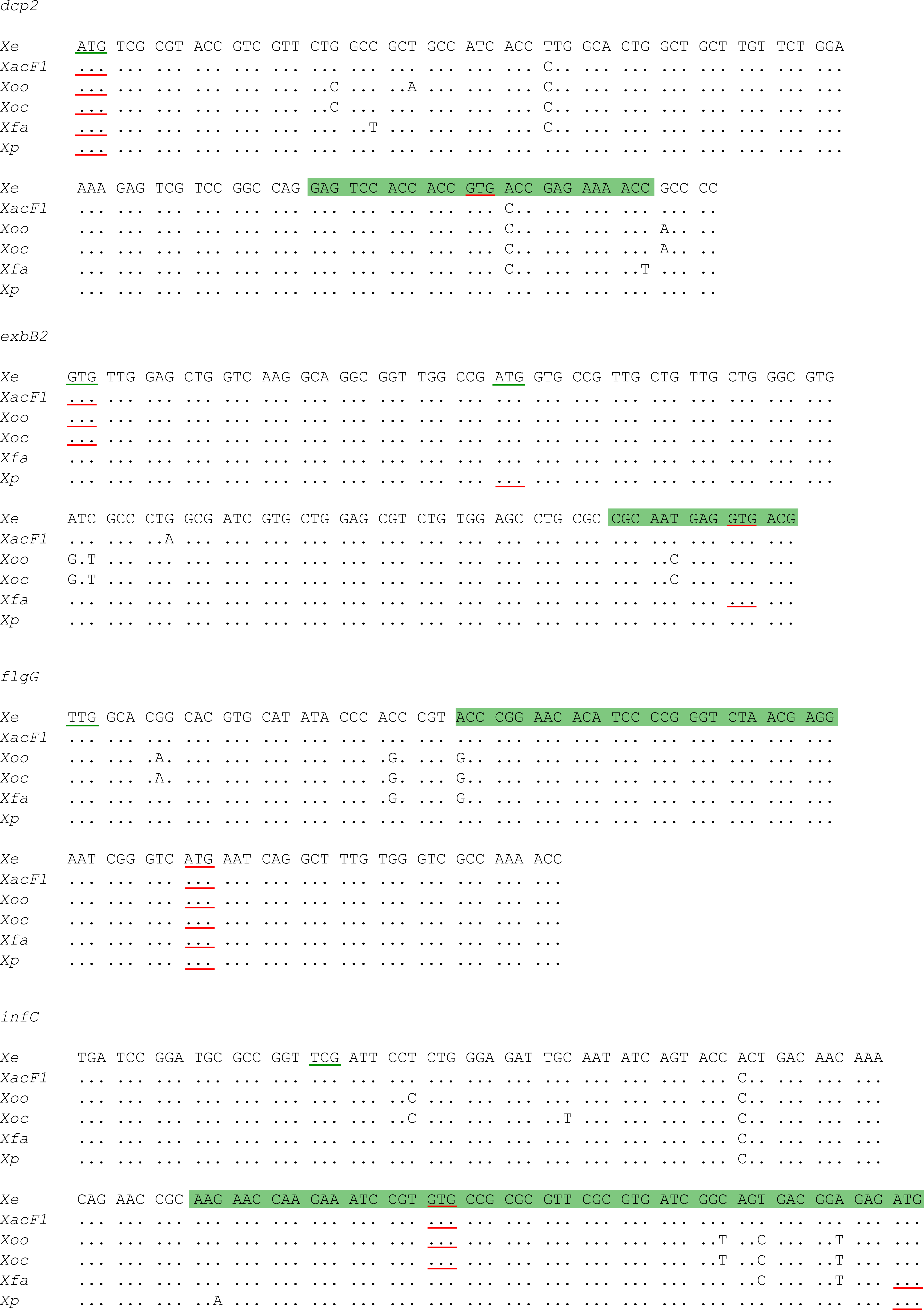

Supplement: Supplementary file 4 — Reannotation of dcp2, exbB2, flgG and infC. Multiple sequence alignment of dcp2, exbB2, flgG and infC homologs of Xe, X. axonopodis pv. citrumelo F1 (XacF1), X. oryzae pv. oryzae KACC10331 (Xoo), X. oryzae pv. oryzicola BLS256 (Xoc), X. fuscans subsp. aurantifolii ICPB 11122 (Xfa), X. perforans 91–118 (Xp). Green: experimentally detected by MS, underlined in red: annotated start codons, underlined in green: possible new start codon. (PNG 836 kb) [file 12864_2017_4041_MOESM4_ESM.png]

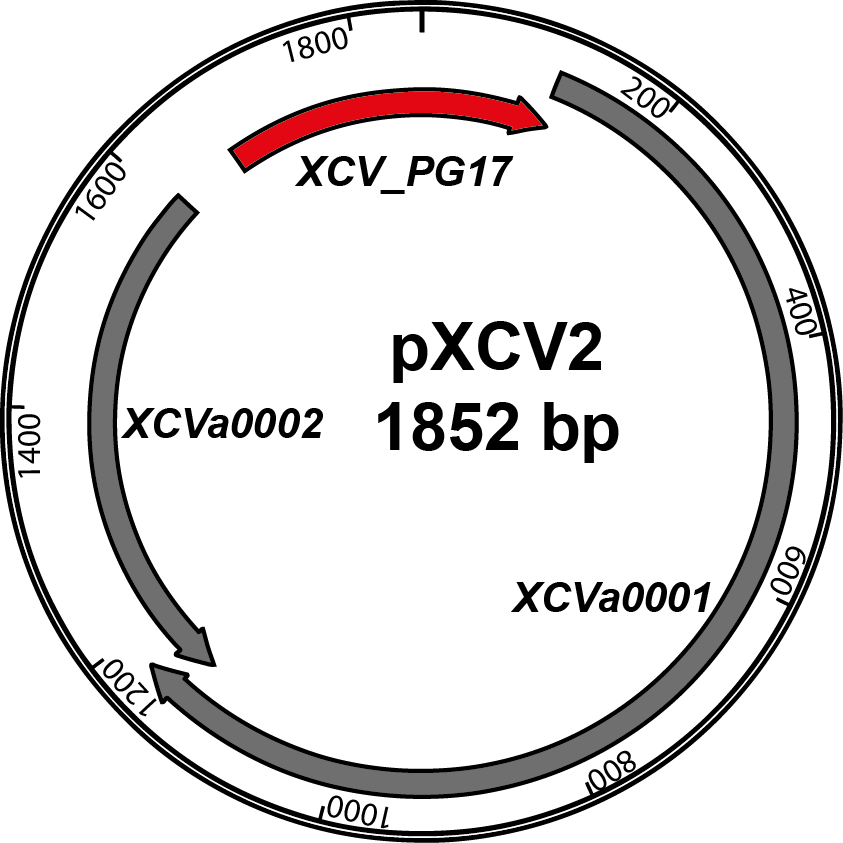

Supplement: Supplementary file 5 — pXCV2 carries a third CDS. Representation of pXCV2 plasmid of Xe 85–10. Grey arrows show position of annotated CDS and the red arrow indicates the position of the newly identified protein-coding CDS. (PNG 88 kb) [file 12864_2017_4041_MOESM5_ESM.png]
