# Supplementary material for: Identification of new protein-coding genes with a potential role in the virulence of the plant pathogen Xanthomonas euvesicatoria
Source: BMC Genomics. 2017 Aug 16;18:625. doi: 10.1186/s12864-017-4041-7 (PMC5559785; doi:10.1186/s12864-017-4041-7)
Supplement: Supplementary file 7 — Oligonucleotides, plasmids and strains used in this study. List of oligonucleotides, plasmids and strains used in this study. (DOCX 45 kb) [file 12864_2017_4041_MOESM7_ESM.docx]

Additional File 7: Oligonucleotides, plasmids and strains used in this study

| **Oligonucleotides used in this study** | | | | | |
| --- | --- | --- | --- | --- | --- |
| **Name** | **Sequence (5’ -> 3’)** | | | **Purpose** | |
| PG02_for | TTTGGTCTCTATTCATTCAGTAGTCGGCCCAAGCT | | | Generation of pBRM-P(*XCV_PG02*) | |
| PG02_rev | TTTGGTCTCTCACCTGGGTGCAGCCTTCCCCTGGC | | |  |  |
| PG06_for | TTTGGTCTCTATTCCCTCTATCGGGCCACGCCCT | | | Generation of pBRM-P(*XCV_PG06*) | |
| PG06_rev | TTTGGTCTCTCACCCTTGCCGAGCGAACCAATGCA | | |  |  |
| PG07_for | TTTGGTCTCTATTCACGCGGCGGCGTGGAGCCAGA | | | Generation of pBRM-P(*XCV_PG07)* | |
| PG07_rev | TTTGGTCTCTCACCGATATCTAAAAGTCGTCGCGC | | |  |  |
| PG13_for | TTTGGTCTCTATTCGTAAGGGAGCGGTGGCGGTGT | | | Generation of pBRM-P(*XCV_PG13*) | |
| PG13_rev | TTTGGTCTCTCACCGAAAGGCTGACGCGGGTTGTC | | |  |  |
| PG17_for | TTTGGTCTCTATTCAAACCCTTGCCTGGCTTTGCT | | | Generation of pBRM-P(*XCV_PG17*) | |
| PG17_rev | TTTGGTCTCTCACCtctgttgcgcttcctcatggc | | |  |  |
| dskA_for | TTTGGTCTCTATTCACTTGCCAATGATATGCCACT | | | Generation of pBRM-P(*dksA*) | |
| dskA_rev | TTTGGTCTCTCACCGTCGCCCTGCTGCTTCTGCAA | | |  |  |
| 1265_for | TTTGGTCTCTATTC gccgtcgatcatccagacgcg | | | Generation of pBRM-P(*XCV1265*) | |
| 1265_rev | TTTGGTCTCTCACC tcccgccgaccaacgccaatg | | |  |  |
| dksA_mut1_for | ACGAAGGATGAGCGTTTCAGGGCTGCTAAAAAACCT | | | Site-directed mutagenesis of pBRM-P(*dksA*) to generate pBRM-P(*dksA GTG1_m_*_t_) | |
| dksA_mut1_rev | TCCCCCGGGGCCCGGGAAAGC | | |  |  |
| dksA_mut2_for | gcagcccccaagtcaaggaaacccgtcgccaag | | | Site-directed mutagenesis of pBRM-P(*dksA*) to generate pBRM-P(*dksA GTG2_mt_*) | |
| dksA_mut2_rev | GGACGCGGTGGTCTTGGAAGC | | |  |  |
| dksA_mut3_for | CTCCCAAGACCAAGTACAAGAGGGTCGAGTACAAGACCGACG | | | Site-directed mutagenesis of pBRM-P(*dksA*) to generate pBRM-P(*dksA GTG3_mt_*) | |
| dksA_mut3_rev | CCGCGCTGGACGGCTTGGACGTCAC | | |  |  |
| 1265_mut1_for | ccgctaagctgcgtagAGGcatcatcttcaacgt | | | Site-directed mutagenesis of pBRM-P(*XCV1265*) to generate pBRM-P(*XCV1265 ATG1_mt_)* | |
| 1265_mut1_rev | ttgcctggtgagcgctgccag | | |  |  |
| 1265_mut2_for | cttccgcagggtctgAGGccgctgatcccgcgg | | | Site-directed mutagenesis of pBRM-P(*XCV1265*) to generate pBRM-P(*XCV1265 ATG2_mt_*) | |
| 1265_mut2_rev | atgtgcagccagatagcgtcc | | |  |  |
| TssFGH1_del_5’_for | TTTGGTCTCTCGACCAGGCATTGCAACACGATC | | | Generation of pOGG2(TssFGH1) | |
| TssFGH1_del_5’_rev | TTTGGTCTCTGTTAGCTCAGTGCGCTCCGGAC | | |  |  |
| TssFGH1_del_3’_for | TTTGGTCTCTTAACGGGCAGAAAACATGGTG | | |  |  |
| TssFGH1_del_3’_rev | TTTGGTCTCTATGGATTCGTCTCTGCCTTG | | |  |  |
| TssFGH2_del_3’_for | TTTGGTCTCTATGGGATCGGTGAGTCGGCCGAATG | | | Generation of pOGG2(TssFGH2) | |
| TssFGH2_del_3’_rev | TTTGGTCTCTTAACCGAGCCGCCCGGTTTCCAG | | |  |  |
| TssFGH2_del_5’_for | TTTGGTCTCTGTTAGTAGCGGTTTCAATC | | |  |  |
| TssFGH2_del_5’_rev | TTTGGTCTCTCGACGATGCAAACATAGAG | | |  |  |
| TssI1_del_5’_for | TTTGGTCTCTCGACAGATGGGACTGCCGAAGGAC | | | Generation of pOGG2(TssI1) | |
| TssI1_del_5’_rev | TTTGGTCTCTGGCATCGCCTCGATGCCC | | |  |  |
| TssI1_del_3’_for | TTTGGTCTCTTGCCGGACCGCCATGTTGCAGAT | | |  |  |
| TssI1_del_3’_rev | TTTGGTCTCTATGGGTTCGGCAAACACATCGCG | | |  |  |
| TssI2_del_5’_for | TTTGGTCTCTCGACGTTGAGGAAGCGCACCACC | | | Generation of pOGG2(TssI2) | |
| TssI2_del_5’_rev | TTTGGTCTCTCCGACGCCTTGGTGCAGGT | | |  |  |
| TssI2_del_3’_for | TTTGGTCTCTTCGGCGAAGCATCCACAGGTGATG | | |  |  |
| TssI2_del_3’_rev | TTTGGTCTCTATGGCAGGCGCAAACGGTGTATCG | | |  |  |
| AvrBs2_del_5’_for | GATTGTCGACATAACTTCCGATCAC | | | pOKI(avrBs2) | |
| AvrBs2_del_5’_rev | GATAAGCTTGATGACCTCGAAAACGCGG | | |  |  |
| AvrBs2_del_3’_for | ACGAAGCTTACCTTCCAATCACGGCTTC | | |  |  |
| AvrBs2_del_3’_rev | CTGTCTAGAGCGATTCCCACCGAGGCGC | | |  |  |
| **Plasmids used in this study** | | | | | |
| **Name** | | | **Feature** | | **Reference** |
| pBRM-P | | | Golden Gatec cloning vector; derivative of pBBR1MCS-5 without lac promoter for gene expression controlled by the own promoter and transcriptionally fused to a 3 xc-Myc epitope-encoding sequence | | [1] |
| pBRM-P(*XCV_PG02*) | | | Derivative of pBRM-P encoding *XCV_PG02* CDS and putative promoter | | This study |
| pBRM-P(*XCV_PG06*) | | | Derivative of pBRM-P encoding *XCV_PG06* CDS and putative promoter | | This study |
| pBRM-P(*XCV_PG07*) | | | Derivative of pBRM-P encoding *XCV_PG07* CDS and putative promoter | | This study |
| pBRM-P(*XCV_PG13*) | | | Derivative of pBRM-P encoding *XCV_PG13* CDS and putative promoter | | This study |
| pBRM-P(*XCV_PG17*) | | | Derivative of pBRM-P encoding *XCV_PG17* CDS and putative promoter | | This study |
| pBRM-P(*dksA*) | | | Derivative of pBRM-P encoding *dksA* and putative promoter | | This study |
| pBRM-P(*dksA GTG1_mt_*) | | | Derivative of pBRM-P(*dksA*) with AGG mutation of the first GTG | | This study |
| pBRM-P(*dksA GTG2_mt_*) | | | Derivative of pBRM-P(*dksA*) with AGG mutation of the second GTG | | This study |
| pBRM-P(*dksA GTG3_mt_*) | | | Derivative of pBRM-P(*dksA*) with AGG mutation of the previously annotated GTG | | This study |
| pBRM-P(*XCV1265*) | | | Derivative of pBRM-P encoding *XCV1265* and putative promoter | | This study |
| pBRM-P(*XCV1265 ATG1_mt_*) | | | Derivative of pBRM-P(*XCV1265*) with AGG mutation of the first ATG | | This study |
| pBRM-P(*XCV1265 ATG2_mt_*) | | | Derivative of pBRM-P(*XCV1265*) with AGG mutation of the previously annotated ATG | | This study |
| pOKI | | | Suicide vector; sacB sacQ mobRK2 oriR6K | | [2] |
| pOKI(*avrBs2*) | | | Derivative of pOKI carrying flanking regions of *avrBs2* | | This study |
| pOGG2 | | | Golden Gate cloning vector, derivative of suicide vector pOK1, sacB sacQ mobRK1 oriR6K | | [3] |
| pOGG2(TssFGH1) | | | Derivative of pOGG2, carrying regions upstream of *XCV2125* and downstream of *XCV2127* | | This study |
| pOGG2(TssFGH2) | | | Derivative of pOGG2, carrying regions upstream of *XCV4238* and upstream of the TS element adjacent to *XCV4236* | | This study |
| pOGG2(TssI1) | | | Derivative of pOGG2 carrying flanking regions of *XCV2133* | | This study |
| pOGG2(TssI2) | | | Derivative of pOGG2 carrying flanking regions of *XCV4217* | | This study |
| ***Xanthomonas euvesicatoria* strains used in this study** | | | | | |
| **Name** | | **Feature** | | | **Reference** |
| 85-10 | | Pepper-race 2; wild type; Rif resistant | | | [4] |
| 85* | | Derivative of 85-10 containing the *hrpG** mutation | | | [5] |
| 85-10Δ*sX13* | | Derivative of 85-10, chromosomal deletion of *sX13* | | | [6] |
| 85-10ΔTssI1ΔTssI2 | | Derivative of 85-10; chromosomal deletion of *XCV2133* and *XCV4217* | | | This study |
| 85-10ΔTssFGH1ΔTssFGH2 | | Derivative of 85-10; chromosomal deletion of *XCV2125* to *XCV2127* and *XCV4236* to *XCV4238* including the adjacent IS-element | | | This study |
| 85-10ΔavrBs2 | | Derivative of 85-10; chromosomal deletion of *avrBs2* | | | This study |
| 85-10ΔavrBs2ΔTssFGH1 ΔTssFGH2 | | Derivative of 85-10; chromosomal deletion of *avrBs2, XCV2125* to *XCV2127* and *XCV4236* to *XCV4238* including the adjacent IS-element | | | This study |
| 85-10Δ*hrcN* | | Derivative of 85-10; chromosomal deletion of *hrcN*; T3SS deficient | | | [7] |

References

[1] C. Lorenz, J. Hausner, D. Büttner, HrcQ provides a docking site for early and late type III secretion substrates from *Xanthomonas*, (2012) e51063.

[2] E. Huguet, K. Hahn, K. Wengelnik, U. Bonas, hpaA mutants of *Xanthomonas campestris* pv. *vesicatoria* are affected in pathogenicity but retain the ability to induce host‐specific hypersensitive reaction, Mol Microbiol 29(6) (1998) 1379-1390.

[3] S. Schulze, S. Kay, D. Büttner, M. Egler, L. Eschen‐Lippold, G. Hause, A. Krüger, J. Lee, O. Müller, D. Scheel, R. Szczesny, F. Thieme, U. Bonas, Analysis of new type III effectors from *Xanthomonas* uncovers XopB and XopS as suppressors of plant immunity, New Phytol 195(4) (2012) 894-911.

[4] U. Bonas, R.E. Stall, B. Staskawicz, Genetic and structural characterization of the avirulence gene avrBs3 from *Xanthomonas campestris* pv. *vesicatoria*, Mol Gen Genet 218(1) (1989) 127-36.

[5] K. Wengelnik, O. Rossier, U. Bonas, Mutations in the regulatory gene *hrpG* of *Xanthomonas campestris* pv. vesicatoria result in constitutive expression of all *hrp* genes, J Bacteriol 181(21) (1999) 6828-6831.

[6] C. Schmidtke, U. Abendroth, J. Brock, J. Serrania, A. Becker, U. Bonas, Small RNA sX13: a multifaceted regulator of virulence in the plant pathogen *Xanthomonas*, PLoS pathogens 9(9) (2013) e1003626.

[7] C. Lorenz, D. Büttner, Functional characterization of the type III secretion ATPase HrcN from the plant pathogen *Xanthomonas campestris* pv. *vesicatoria*, J Bacteriol 191(5) (2009) 1414-1428.
